# Regional Differences in Antifungal Susceptibility of the Prevalent Dermatophyte *Trichophyton rubrum*

**DOI:** 10.1007/s11046-020-00515-z

**Published:** 2020-12-12

**Authors:** Y. Jiang, W. Luo, P. E. Verweij, Y. Song, B. Zhang, Z. Shang, A. M. S. Al-Hatmi, S. A. Ahmed, Z. Wan, R. Li, G. S. de Hoog

**Affiliations:** 1 Department of Dermatology, The Affiliated Hospital, Guizhou Medical University, Guiyang, China; 2 Department of Medical Microbiology, Radboud University Medical Center, Center of Expertise in Mycology Radboudumc/CWZ, Nijmegen, The Netherlands; 3 Department of Dermatology, Peking University First Hospital, Beijing, China; 4 Research Center for Medical Mycology, Peking University, Beijing, China; 5 National Clinical Research Center for Skin and Immune Disease, Beijing, China; 6 School of Public Health, Guizhou Medical University, Guiyang, China; 7 Department of Immunology, Basic Medical School, Guizhou Medical University, Guiyang, China; 8 Faculty of Medical Laboratory Sciences, University of Khartoum, Khartoum, Sudan; 9 Ministry of Health, Directorate General of Health Services, Ibri, Oman

**Keywords:** Dermatology, Antifungal agents, Drug resistance, Terbinafine, *Trichophyton*

## Abstract

In vitro susceptibility testing for *Trichophyton rubrum* has shown resistance to terbinafine, azoles and amorolfine, locally, but epidemiological cutoffs are not available. In order to assess the appropriateness of current first-line antifungal treatment for *T. rubrum* in China, we characterized antifungal susceptibility patterns of Chinese *T. rubrum* strains to nine antifungals and also described the upper limits of wild-type (WT) minimal inhibitory concentrations (MIC) (UL-WT) based on our study and another six studies published during the last decades. Sixty-two clinical isolates originating from seven provinces in China were identified as *T. rubrum *sensu stricto; all Chinese strains showed low MICs to eight out of nine antifungal drugs. Terbinafine (TBF) showed the lowest MICs of all antifungal classes tested in both the Chinese and global groups, with a 97.5% UL-WT MIC-value of 0.03 mg/L. No non-WT isolates were observed for TBF in China, but were reported in 18.5% of the global group. Our study indicated that TBF was still the most active drug for Chinese *T. rubrum* isolates, and all strains were within the WT-population. TBF therefore remains recommended for primary therapy to dermatophytosis caused by *T. rubrum* in China now, but regular surveillance of dermatophytes and antifungal susceptibility is recommended.

**Supplementary Information:**

The online version of this article (10.1007/s11046-020-00515-z) contains supplementary material, which is available to authorized users.

## Introduction

*Trichophyton rubrum* has been among the most prevalent dermatophytes causing tinea pedis and tinea unguium since the early twenty-first century [1]. The global predominance of *T. rubrum* suggests that this species has a significantly higher capacity of transmission than other anthropophilic dermatophytes [2]. Numerous authors have noted, as confirmed by whole-genome sequencing [3], that *T. rubrum* is clonal with a highly conserved gene content, low levels of variation, and little evidence of recombination. On the Indian subcontinent, the species is still common [4–6], but seems gradually to be replaced by members of the *T. mentagrophytes* group [4, 5, 7], thus having a similar fate as the classical, disappearing dermatophytes *Epidermophyton floccosum* and *Microsporum audouinii* [1]. One of the main characteristics promoting global spread is the low virulence of *T. rubrum* [8]. Its mild, hardly noticeable cutaneous infections with transmission via skin scales released from mild hyperkeratosis does not interfere with transmission-enhancing interaction of human hosts. Infections only are more serious in CARD9-deficient patients or in those with dysfunctional cellular immunity, e.g., with cirrhosis, AIDS, hematological malignancies or solid organ transplants [9–11].

Acquired resistance, as observed in numerous fungi, is however a concern. *T. rubrum* is regarded to have limited capacity to develop resistance to terbinafine even after prolonged exposure [12], but it has been proven that *T. rubrum* can develop resistance to azoles, amorolfine and terbinafine after prolonged exposure to sub-inhibitory concentrations of these drugs [13–18]. Antifungal drug resistance may contribute to treatment failure and lead to persistent and chronic infections [13, 14]. The first report on *T. rubrum* exhibiting resistance to terbinafine was published in 2003 [15], followed by reports from the Americas, Europe and Asia [16–18]. Ebert et al. [19] indicated that resistance of *T. rubrum* for terbinafine was as high as 44%, although lower than that of *T. mentagrophytes* group in India. This study also showed that the systemic antifungal drug terbinafine, first-line drug for dermatophytosis, had lost its in vitro activity in most parts in India [19].

It has been speculated that the resistance in dermatophytes might be related to drug exposure [5, 19]. The management of over-the-counter drug use in China is similar to that in India, but resistance of *T. rubrum* has not systematically been reported from China. No clinical break points (CBP) or epidemiological cutoffs (ECV) are available to guide antifungal treatment and classify resistance in *T. rubrum.* The Guizhou Province, located in the southwest of China, is humid and mountainous, with poor transportation and backward economy. Local people and even doctors in basic hospitals pay insufficient attention to dermatophytosis and have poor awareness of diagnosis and treatment, whether it will lead to difference in drug susceptibility of *T. rubrum* in Guizhou region? Based on that, we compared clinical isolates from Guizhou Province with six regions in China in terms of genomic diversity of strains and in vitro susceptibility to a panel of antifungal drugs. In order to investigate potential trends in drug resistance of *T. rubrum*, we also reviewed available MIC values from published literature and describe wild-type (WT) MIC distributions of nine drugs for *T. rubrum* according to the criteria used by CLSI.

## Materials and Methods

### Strains

Sixty-two clinical *T. rubrum* strains from seven provinces in China were available for testing; geographic and clinical data are given in supplementary Table S-1. Thirty-one isolates cultured from tinea pedis were obtained from the Research Center for Medical Mycology at Peking University: five originated from Guangzhou, Sichuan, Ningxia, Wuhan and Jilin, respectively, and six from Xinjiang. Another 31 clinical isolates were obtained from the Department of Dermatology of Guizhou Medical University of China; of these, five were isolated from tinea pedis, and six others from tinea cruris, tinea corporis, tinea capitis, tinea faciei and tinea manuum, respectively; the remaining 20 strains were from tinea unguium. All strains were isolated during the period 2016–2019.

### Identification

Identification of isolates was done by phenotype [20] and confirmed by rDNA internal transcribed spacer regions (ITS) sequencing. Briefly, isolates were subcultured on Sabouraud’s Glucose Agar (SGA, homemade) incubated at 28 °C for one week. DNA extraction was by the cetyltrimethylammonium bromide (CTAB) method [21]. ITS of the rDNA operon was amplified with primers ITS5 and ITS4 according to in Jiang et al. [21, 22]. PCR products were sequenced by TSINGKE Biological Technology (Beijing, China). GenBank accession numbers for new sequences are given in supplementary Table S-1. For global comparison, 87 reference sequences were retrieved from GenBank, including *T. rubrum* (*n* = 76), *T. violaceum* (*n *= 4), *T. soudanense* (*n* = 4), with *T. verrucosum* (*n* = 3) as outgroup. Alignment was done with MUSCLE using MEGA v6.0 [23], and Bayesian inference analyses (BI) were performed using MRBAYES v3.1.2 [24].

### Antifungal Susceptibility

A panel of nine commonly used topical or systemic antifungal agents (abbreviations according to de Hoog et al. [20]) were tested by the broth microdilution technique of Clinical and Laboratory Standards Institute (CLSI) protocol M38-A3 [25], as follows: luliconazole (LLCZ; Higher Biotech Co, Shanghai, China), amorolfine (AMF; Sigma Aldrich, St. Louis, USA), terbinafine (TBF; CFDA Co., Beijing, China), itraconazole (ITZ; CFDA), bifonazole (BFZ; CFDA), ketoconazole (KTZ; CFDA), miconazole (MCZ; CFDA), fluconazole (FCZ; Sigma Aldrich) and naftifine (NAF; CFDA). Stock solutions of all drugs were prepared in dimethyl sulfoxide (DMSO) at a concentration of 2 mg/mL (except FCZ, which was dissolved in distilled water at final concentration 102.4 mg/mL). Drug stock solutions were diluted in RPMI 1640 medium buffered with 3-N-(morpholino)propanesulfonic acid (MOPS), in twice the final concentration followed by addition of equal volumes of the pre-adjusted inoculum of fungal isolates, in 96-well microtiter plates. Final concentrations of the antifungals tested ranged from 0.125 to 64 μg/mL for FCZ, 0.0078 to 4 μg/mL for KTZ, 0.004 to 2 μg/mL for BFZ, 0.002 to 1 μg/mL for ITZ, MCZ and AMF, 0.00025 to 0.125 μg/mL for TBF, 0.001 to 0.5 μg/mL for NAF and LLCZ. Drug plates were stored at − 70 °C.

### Inoculum and Quantification

Strains were subcultured from primary SGA plates to potato dextrose agar (PDA) to induce conidiation. Plates were incubated at 28 °C for 9–14 days. Conidia were collected by gently flushing 5 mL phosphate buffer saline (pH = 7.4) on colonies and aspirating the suspension into a sterile collection tube. Suspensions were counted on a hemocytometer and diluted in RPMI 1640 to the desired concentration of 1 × 10^3^ ~ 3 × 10^3^ CFU/mL.

### Reading of Results

Microdilution plates were incubated at 35 °C and visually read after 5–7 days. Endpoint MICs for azoles and TBF were considered when prominent inhibition (approximately 80%) was reached compared to the control wells, while for NAF and AMF 100% growth inhibition was required. Ranges and geometric means (GMs) of the MICs were determined for each group and drug after 6 days. If no growth was observed or growth was inadequate, incubation was extended to 7 days. *Candida parapsilosis* (ATCC 22019) and *T. mentagrophytes* (ATCC MYA 4439) were included as quality control strains. All experiments were performed using two independent replicates on different days.

### Statistics

Statistical analysis was performed by Mann–Whitney *U* and Kruskal–Wallis tests (Student’s *t* test) using SPSS software v21. One-way analysis of variance (ANOVA) was used to compare the geometric mean (GM) MICs between the groups and within distinct geographic areas. *P* values of < 0.05 were considered significant. ECV values were computed by the Microsoft Excel spreadsheet calculator ECOFFinder XL 2010 v2.1 (https://clsi.org/), which follows a methodology established by Turnidge et al. [26]

### Literature Search

Keywords “Dermatophytes”, “*Trichophyton rubrum*”, “Antifungal susceptibility”, “Resistance”, “Minimum inhibitory concentration” and “Geometric mean (GM)” were used in a PubMed and CNKI (China National Knowledge Infrastructure) to screen English and Chinese literature for research articles and reviews published from 2010 to 2020. In addition, CLSI M38-A2 broth microdilution protocol was considered as a restrictive condition for literature review.

## Results

### Identification

**Fig. 1:**
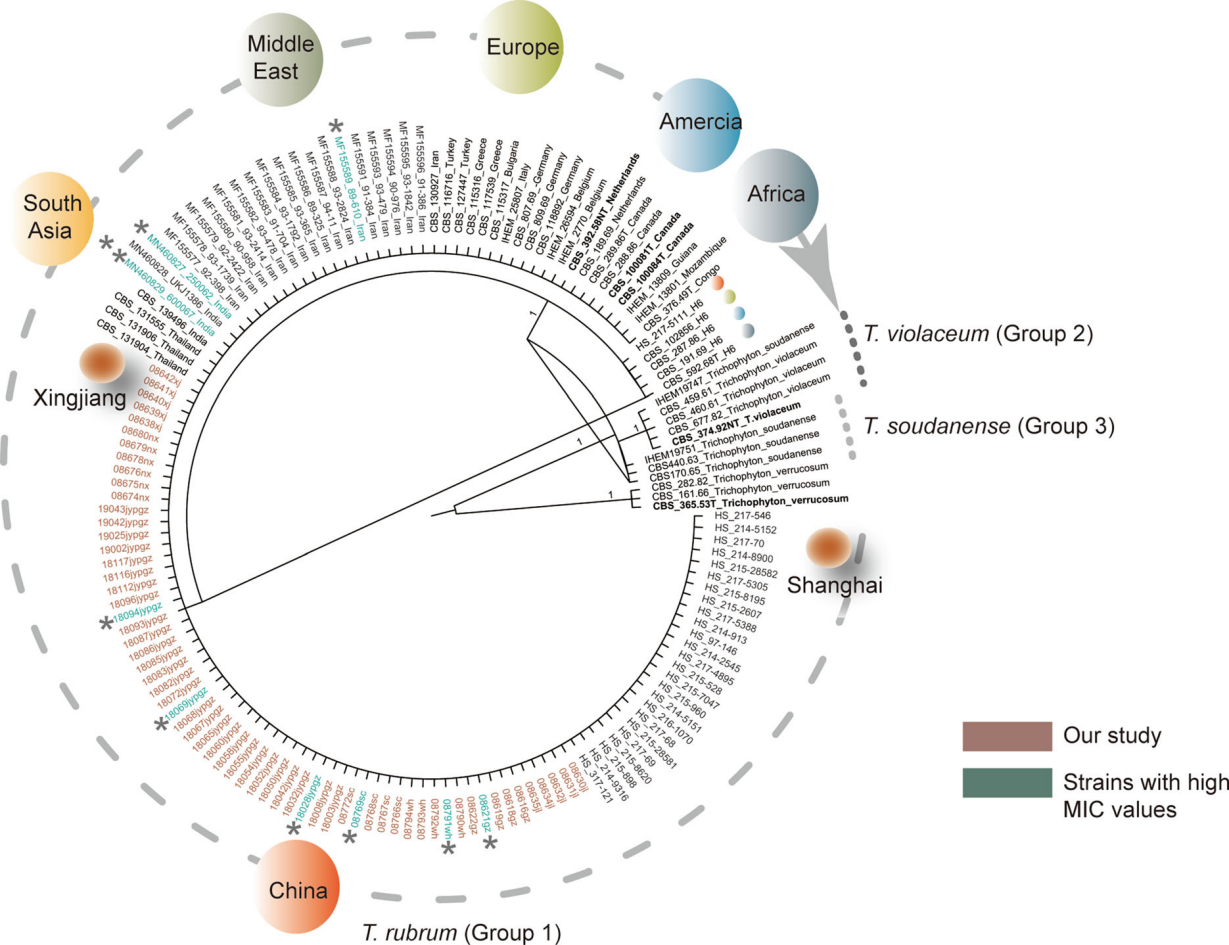
Single-locus representation of ITS sequences in *Trichophyton rubrum* complex. Bootstrap (BPP > 0.95) from BI analyses is shown along the branches. *T. rubrum* monophyletic clades (Group 1) are color-coded according to geographic region. Trees are rooted with *T. verrucosum.* The strains in this study are marked in red, and the strains with high MIC values for FCZ or ITZ or TBF are marked in blue. Asterisks represent numbers of insensitive drugs. (Color figure online)

*T. rubrum* is reliably identified by the rDNA ITS barcoding marker [27, 28]. Phylogenetic analysis resolves species boundaries between the closely related siblings in the *T. rubrum* complex (Fig. 1). In the alignment (555 bp including gaps), three groups differing by single nucleotide polymorphisms (SNPs) were revealed. Group 1 (*T. rubrum *sensu stricto) comprised two clusters matching with Haplotypes 5 and 6 [27]. H5 comprised 134 identical isolates and included the neotype strain CBS 392.58 (Group 1A). All strains from our study clustered into this subclade and revealed 100% sequence similarity. Five strains (Group 1B), one of which was from China, deviated by a single SNP matching haplotype H6 [27]. Two remaining clusters in the *T. rubrum* complex contained reference strains of *T. violaceum* (Group 2, 7 bp distance) and *T. soudanense* (Group 3, 4 bp distance).

### Antifungal Susceptibility

**Fig. 2:**
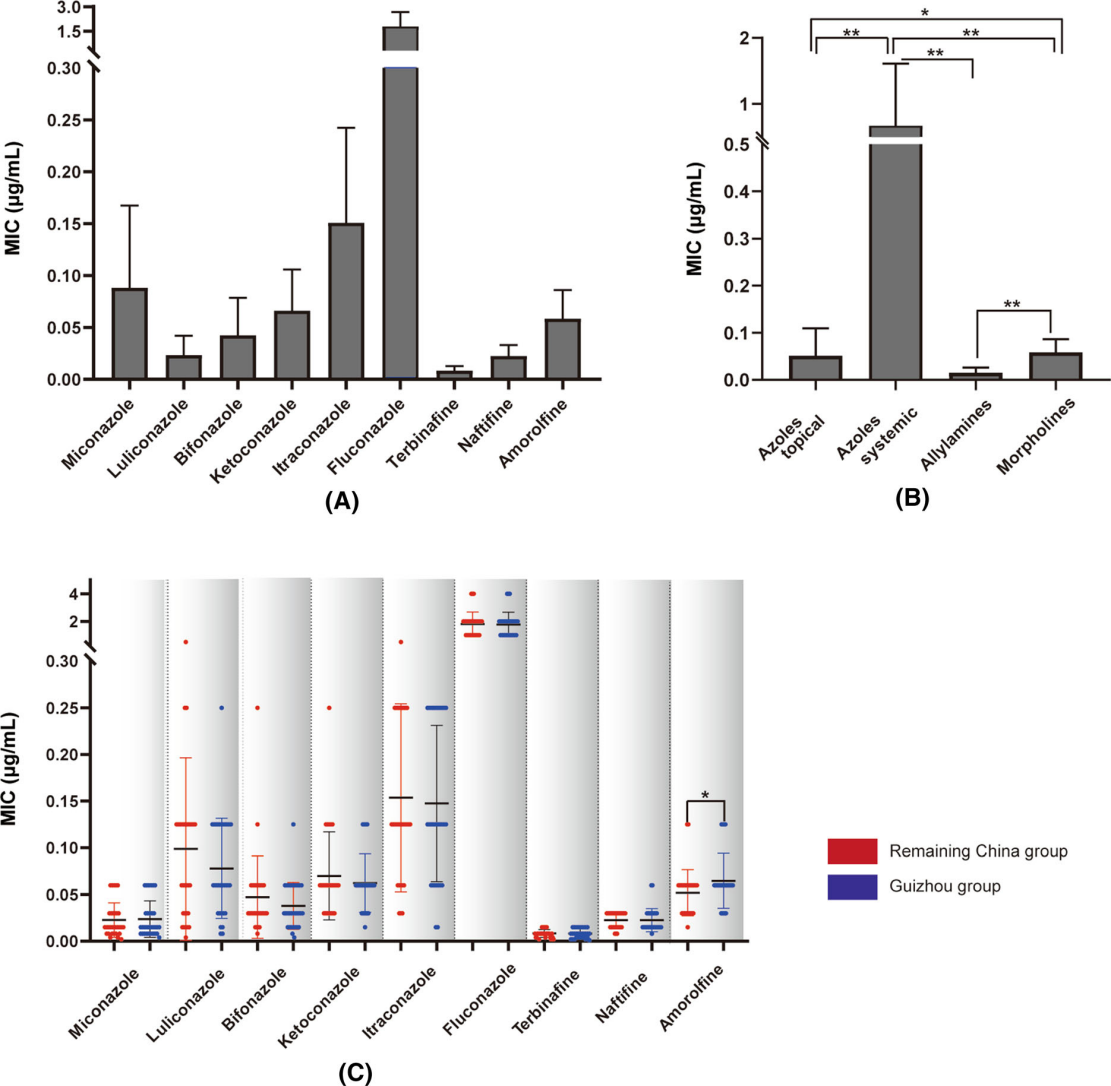
Comparison of MIC values of 62 T*. rubrum* strains. (**A**) Box plot of nine antifungals tested. (**B**) Comparison of four categories of drugs. (**C**) Comparison of Guizhou group (red) with remaining China group (blue). **(*P* < 0.01), *(*P* < 0.05). (Color figure online)

MIC ranges, geometric means (GMs) of MICs and MIC50/MIC90 ratios were obtained for nine antifungals and 62 isolates applying the CLSI protocol (Table 1, Fig. 2a). Among nine drugs, TBF was the most active antifungal drug against all *T. rubrum* strains, with lowest MICs (GM = 0.00688 μg/mL, MIC50 = 0.008 μg/mL, MIC90 = 0.015 μg/mL; *P* < 0.01). LLCZ also was active with low MIC values (GM = 0.0169 μg/mL, MIC50 = 0.015 μg/mL, MIC90 = 0.06 μg/mL), but the difference with NAF and MCZ was not statistically significant (*P* > 0.05).

**Table 1:** MIC values (μg/mL) of nine antifungal agents against *Trichophyton rubrum* determined according to geographic location and drug class

Group (no. of isolates)	Parameter	Drugs (μg/ml)								
		Azoles						Allylamines		Morpholines
		Topical			Systemic			Systemic or topical		Topical
		LLCZ	BFZ	MCZ	KTZ	FCZ	ITZ	TBF	NAF	AMF
Guizhou (31)	Range	0.004–0.06	0.004–0.06	0.008–0.25	0.015–0.125	1–4	0.015–0.25	0.001–0.015	0.008–0.06	0.03–0.125
	GM	0.017514	0.030165	0.058533	0.055229	1.59927	0.118125	0.006565	0.020102	0.059061
	MIC50	0.015	0.03	0.06	0.06	2	0.125	0.008	0.015	0.06
	MIC90	0.06	0.06	0.125	0.125	2	0.25	0.015	0.03	0.125
Remaining China (31)	Range	0.002–0.06	0.008–0.25	0.004–0.5	0.03–0.25	1.00–4	0.03–0.5	0.002–0.015	0.008–0.03	0.015–0.125
	GM	0.016344	0.036861	0.062711	0.059217	1.635438	0.12369	0.00721	0.020642	0.047041
	MIC50	0.015	0.03	0.06	0.06	2	0.125	0.008	0.03	0.06
	MIC90	0.06	0.06	0.125	0.125	2	0.25	0.015	0.03	0.06
Total (62)	Range	0.002–0.06	0.004–0.25	0.004–0.5	0.015–0.25	1.00–4	0.015–0.5	0.001–0.015	0.008–0.06	0.015–0.125
	GM	0.016919	0.033345	0.060586	0.057188	1.617256	0.120875	0.00688	0.02037	0.05271
	MIC50	0.015	0.03	0.06	0.06	2	0.125	0.008	0.015	0.06
	MIC90	0.06	0.06	0.125	0.125	2	0.25	0.015	0.03	0.125
		Aozle Topical			Aozle Systemic			Allylamines		**Morpholines**
	Range		0.002–0.5			0.015–4		0.001–0.006		0.015–0.125
	GM		0.03245			0.2236		0.01183		0.05271
	MIC50		0.03			0.125		0.015		0.06
	MIC90		0.125			2		0.03		0.125

MIC, minimal inhibitory concentration; GM, geometric mean; AMF amorolfine; BFZ, bifonazole; FCZ, fluconazole; ITZ, itraconazole; KTZ, ketoconazole; LLCZ, luliconazole; MCZ, miconazole; NAF naftifine; TBF, terbinafine

The nine drugs belong to four categories, and MIC values were compared in the 62 T*. rubrum* strains accordingly. Significant differences between antifungal classes were observed (Fig. 2b). Azoles were divided into a topical and a systemic category, respectively. The systemic azoles included KTZ, ITZ and FCZ, while the topical azoles included LLCZ, BFZ and MCZ. MIC values of the systemic azoles were significantly higher than those of the topically administered compounds (GM 0.2236 *vs.* 0.03245 μg/mL; *P* < 0.001). Allylamines, including TBF and NAF, which can be used systemically or topically, were associated with lowest MICs (*P* < 0.001). The morpholine derivatives included the single drug AMF, which is used only topically. AMF was active against all *T. rubrum* strains with low MICs, GMs being just slightly higher than that of the topical azole category (GM: 0.05271 vs. 0.03245 μg/mL, *P* < 0.05).

For a third comparison of MIC values, we separated the *T. rubrum* strains geographically, i.e., a Guizhou group and those from remaining Chinese provinces. There was no difference in drug susceptibility between the two groups for the eight drugs; only for AMF, the 31 strains from Guizhou showed slightly higher MIC values than those from remaining China (GM MIC values: 0.059 vs. 0.047 μg/mL, *P* < 0.05) (Fig. 2C).

### Literature Review and UL-WT Determination

**Fig. 3:**
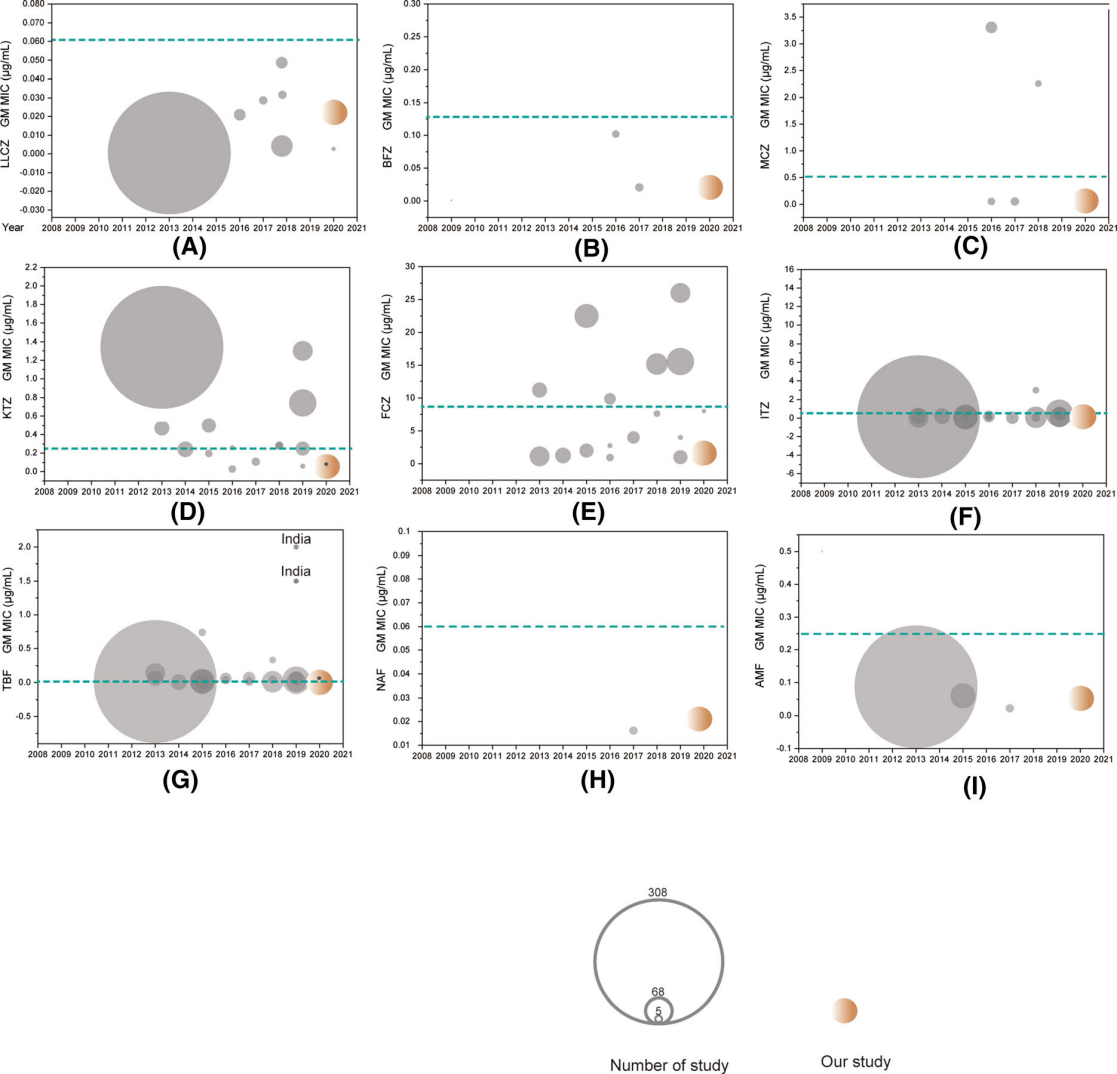
Comparison of MIC values for nine antifungal drugs in *T. rubrum* strains from 2010 to 2020. (**A**–**I**). LLCZ, BFZ, MCZ, KTZ, ITZ, TBF, NAF, AMF. The circle represents each study, and the center of the circle corresponds to the GM value; our study is highlighted in red. The blue line represents UL-WT MIC values for every drug. (Color figure online)

**Fig. 4:**
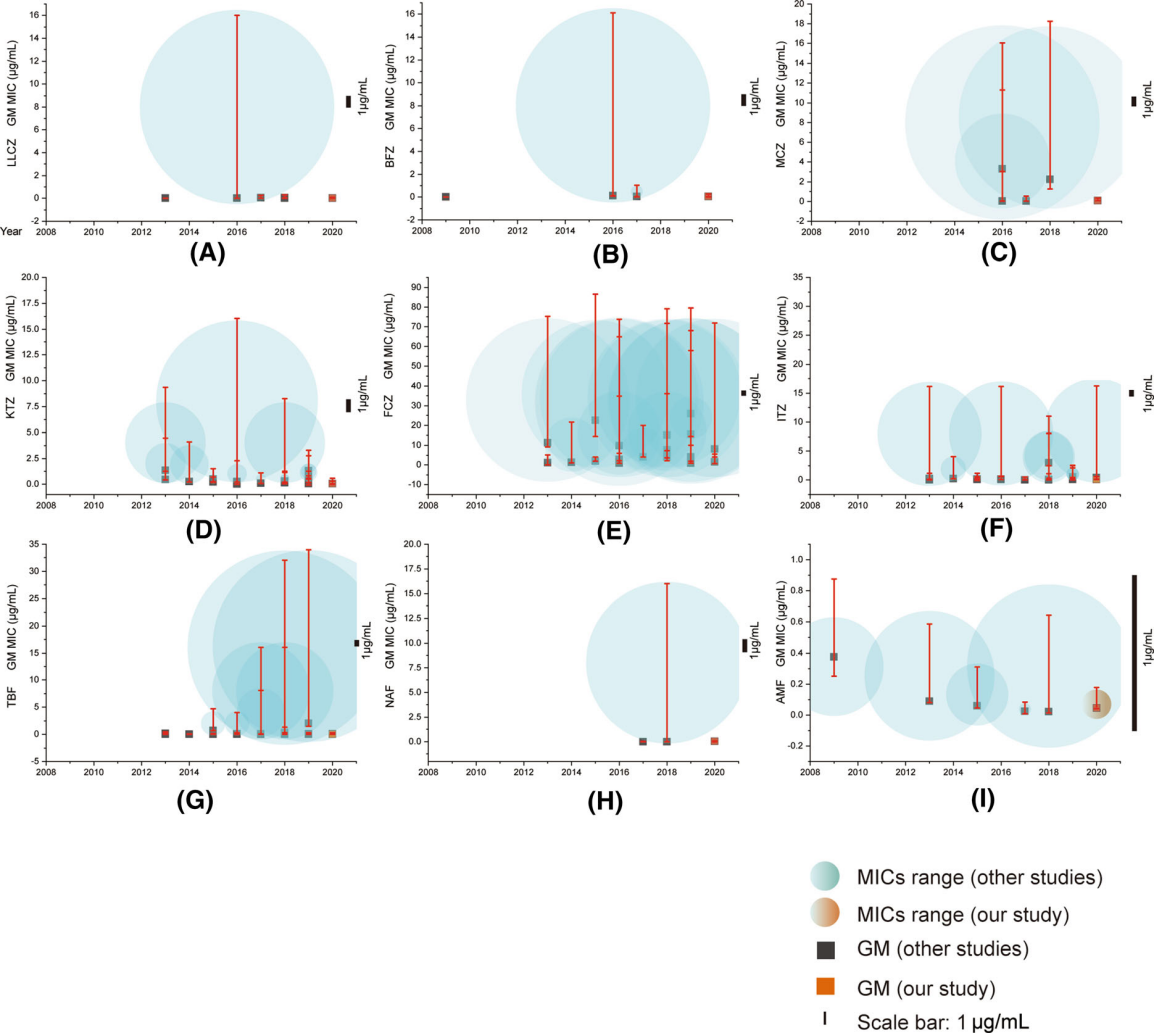
Comparison of MIC values for nine antifungal drugs in *T. rubrum* strains from 2010 to 2020. (**A**–**I**). LLCZ, BFZ, MCZ, KTZ, ITZ, TBF, NAF, AMF. The spherical shape represents the range of MIC values for each study, the error bar is shown in red, and the black spot represents the GM value. Scale bar: 1 μg/mL. (Color figure online)

We subsequently compared published susceptibility data (MIC range and GM) of *T. rubrum* for the nine drugs during the last decade (Table 2, Figs. 3, 4). Since the year 2000, a total of 26 AFST studies comprising 1153 isolates have been carried out with the CLSI M38A2 protocol [29], including 7 in China [30–35]; 9 in India; 6 in Iran; and one each in the USA., Germany, Brazil and Japan [6, 18, 19, 36–50]. The number of *T. rubrum* strains included in the studies ranged from 5 to 308; inoculum sizes ranged from 10^3^ to 10^4^ CFU/mL; most studies (13/26) incubated at 35 °C for a period of 4–7 days. Twenty studies were published during the last five years, with the most frequently studied drugs being ITZ, FCZ, KTZ and TBF. In 26 studies, the largest disparity in GM MIC values was observed with TBF, values differing in nine dilution steps (0.004–2 μg/mL; Figs. 3g, 4g), followed by ITZ (equal to 6 dilution steps, 0.03–3.0 μg/mL; Figs. 3f, 4f). For FCZ, a high GM averaged over all studies was noted (4.56 μg/mL), with a maximum disparity of 5 dilution steps (0.96–25.99 μg/mL; Fig. 3e). Only TBF showed a non-normal distribution, representing India's spheres (2019

**Table 2:** Comparative susceptibility data with MIC ranges and GM (μg/mL) of *T. rubrum* from studies in China and other countries with CLSI M38-A2

Number^†^	Location (no. of isolated)	Year	Incubationtime (day)	Inoculum size (CFU/ml)	Temp. (ºC)	MIC^††^ (μg/ml)	Azoles					
							Topical			Systemic		
							LLCZ	BFZ	MCZ	KTZ	FCZ	ITZ
1	Germany(7)	2009	4–5 days	6.25 × 10^3^	35	Range	–	< 0.0005	–	–	–	–
						GM	–	< 0.0005	–	–	–	–
2	Tianjing (50)	2013	4–7 days	2–3 × 10^3^	35	Range	–	–	–	–	1–4	0.03–0.12
						GM	–	–	–	–	1.117	0.055
3	Brazil (37)	2013	7 days	2–4 × 10^4^	28	Range	–	–	–	≤ 0.031–4	2- ≥ 64	≤ 0.031–1
						GM	–	–	–	0.46	11.2	0.22
4	USA (308)	2013	4 days	1–3 × 10^3^	30	Range	0.00012–0.0025	–	–	0.062–8		0.015–16
						GM	0.00022	–	–	1.34		0.037–0.247
5	India (40)	2014	7 days	0.5–5 × 10^4^	28	Range	–	–	–	0.01–3.84	0.16–20.48	0.03–3.84
						MIC50	–	–	–	0.24	1.28	0.24
6	Japan (62)	2015	4 days	0.5 × 10^3^	35	Range	–	–	–	–	–	0.015–0.25
						MIC50	–	–	–	–	–	0.12
7	India (35)	2015	4 days	1–3 × 10^3^	37	Range	–	–	–	0.25 -1	0.125—2	–
						GM	–	–	–	0.5	2	–
8	India (18)	2015	4–5 days	2–6 × 10^3^	35	Range	–	–	–	0.0156–0.5		0.0156–1
						GM	–	–	–	0.1954		0.1918
9	Iran (60)	2015	3–5 days	1–3 × 10^3^	–	Range	–	–	–	–	8–64	0.16–0.5
						GM	–	–	–	–	22.49	0.088
10	Shanghai (55)	2016	96 h	1–5 × 10^4^	28	Range	–	0.03– > 16	0.03–16	0.03- > 16	0.125- > 64	0.03- > 16
						GM	–	0.11	0.05	0.03	0.96	0.13
11	Iran (13)	2016	4 days	0.4–5 × 10^4^	30	Range	–	–	–	0.06–2	0.5–32	0.03–0.5
						GM	–	–	–	0.26	2.75	0.13
12	Iran (29)	2016	4 days	0.5–3 × 10^3^	35	Range	0.016– 16	–	0.25–8	–	4–64	0.063–0.5
						GM	0.02	–	3.31	–	9.9	0.18
13	Beijing (20)	2017	5–7 days	1–3 × 10^3^	35	Range	0.015–0.25	0.015–1	0.015–0.5	0.03–1	–	–
						GM	0.036	0.035	0.048	0.109	–	–
14	India (5)	2017	–	1–3 × 10^3^	28–30	Range	–	–	–	–	–	0.03–0.06
						GM	–	–	–	–	–	0.039
15	India (29)	2017	–	–	–	Range	–	–	–	–	0.03–16	0.03–0.5
						MIC50	–	–	–	–	4	0.03
16	Wuhan (17)	2018	5–7 days	1–5 × 10^4^	28	Range	< 0.031	–	1–16	0.18–1	4–64	0.5–8
						GM	< 0.031	–	2.26	0.28	7.67	3.01
17	Iran (54)	2018	4 days	1–3 × 10^3^	35	Range	0.0005 -0.002	–	–	–	8–64	0.03–0.25
						GM	0.0004	–	–	–	15.19	0.077
18	Iran (20)	2018	4 days	–	35	Range	–	–	–	0.06–8	–	0.01–8
						GM	–	–	–	0.28	–	0.05
19	India	2018	5 days	0.5–5 × 10^4^	28	Range	0.0312–0.25	–	–	0.0625–1	2–32	0.015–1
	(35)					GM	0.0509	–	–	0.13	4.08	0.09
20	Henan (50)	2019	7 days	1–3 × 10^3^	28	Range	–	–	–	0.5–2	16–32	0.063–0.125
						GM	–	–	–	1.3013	25.992	0.0848
21	Shengzhen (68)	2019	4–5 days	0.5–2.5 × 10^3^	35	Range	–	–	–	0.25–2	1- > 64	0.06–2
						GM	–	–	–	0.744	15.518	0.535
22	India (36)	2019	4 days	1–3 × 10^4^	35	Range	–	–	–	0.06–1	0.125–1	0.125–2
						MIC90	–	–	–	0.25	1	0.5
23	India (13)	2019	5–7 days	1–3 × 10^3^	35	Range	–	–	–	0.125–0.5	2–64	0.125–2
						MIC50	–	–	–	0.06	4	0.125
24	India (18)	2019	–	–	–	Range	–	–	–	–	–	0.0625–1
						GM	–	–	–	–	–	0.18
25	Iran (10)	2020	4 days	1–3 × 10^3^	35	Range	0.0005–0.004	–	–	0.03–0.5	4- 64	0.03- 16
						GM	0.0005	–	–	0.0604	8	0.1871
26	Beijing (62)	2020	5–7 days	1–3 × 10^3^	35	Range	0.002–0.06	0.004–0.25	0.004–0.25	0.015–0.25	1.00–4	0.015–0.5
						GM	0.016919	0.033345	0.060586	0.057188	1.617256	0.120875

Number^†^	Allylamines		Morpholines	References
	Systemic or topical		Topical	
	TBF	NAF	AMF	
1	–	–	0.125–0.5	[36]
	–	–	0.375	
2	0.04–0.32	–	–	[30]
	0.135	–	–	
3	≤ 0.031–0.062	–	–	[37]
	0.06	–	–	
4	0.004–0.25	–	0.008–0.5	[38]
	0.0194	–	0.0883	
5	0.001–0.08	–	–	[39]
	0.005	–	–	
6	0.004–0.03	–	0.015–0.25	[40]
	0.015	–	0.06	
7	0.001–0.008	–	–	[41]
	0.004	–	–	
8	0.0313–4	–	–	[42]
	0.7378	–	–	
9	0.004–0.125	–	–	[43]
	0.017	–	–	
10	0.0075- > 4	–	–	[31]
	0.04	–	–	
11	–	–	–	[44]
	–	–	–	
12	0.008–0.25	–	–	[45]
	0.06	–	–	
13	0.004–0.03	0.004–0.03	0.015–0.06	[32]
	0.012	0.013	0.023	
14	0.03–8.0	–	–	[46]
	0.0863	–	–	
15	0.015–16	–	–	[6]
	0.063	–	–	
16	0.031–1	–	–	[33]
	0.33	–	–	
17	0.004–0.06	–	–	[47]
	0.009	–	–	
18	0.003- > 32	–	–	[18]
	0.04	–	–	
19	0.015–16	0.0312–16	0.007–0.625	[5]
	0.05	0.007	0.02	
20	0.002–0.008	–	–	[34]
	0.0045	–	–	
21	0.016–0.064	–	–	[35]
	0.032	–	–	
22	0.008–0.256	–	–	[48]
	0.064	–	–	
23	0.5–32	–	–	[49]
	2	–	–	
24	0.0312–8	–	–	[19]
	1.1866	–	–	
25	0.003–0.25	–	–	[50]
	0.0678	–	–	
26	0.001–0.015	0.008–0.06	0.015–0.125	Our study
	0.00688	0.02037	0.05271	

^†^UL-WT was determined for seven studies (13, 14, 20, 23, 24, 25, 26)

^††^Several studies (5, 6, 15, 23) indicated MIC50 values rather than GM MIC values; one study (22) indicated MIC90 values

“–”: Data was not available

) deviating significantly from the others (Fig. 3g).

In addition to our own data, six published studies presented the distribution of MICs values for nine antifungal drugs against *T. rubrum*, including two studies form China, three studies from India and one from Iran. We compared to the upper limit of wild-type MIC (UL-WT) distribution of *T. rubrum* between China and the global data set (Table 3). The number of strains tested exceeded 100 for KTZ, ITZ and TBF. Global data were not available for BFZ, MCZ, NAF and AMF. Both the 95% and 97.5% MICs were calculated to determine the wild-type MIC (UL-WT). For the antifungals which showed a multimodal, non-symmetrical or truncated MIC frequency distribution, the MIC95 and MIC97.5 were documented as UL-WT. Highest 97.5% UL-WT were observed for FCZ (8 mg/L), whereas lowest value of 0.03 mg/L was found for TBF. The MICs comprising > 97.5% of the model populations were the same in both groups, as follows: 0.0312 for TBF; 0.0625 for NAF and LLCZ; 0.125 for BFZ; 0.25 for KTZ and AMF; 0.5 for MCZ and ITZ; 8 for FCZ. Highest percentage of isolates above the upper limit of wild-type MIC (UL-WT) were observed for KTZ (global: 34.2%; China: 38.6%). Low percentages of isolates above UL-WT were observed for LLCZ (global: 3.3%; China: 3.7%) and BFZ (global: 3.3%; China: 3.7%). The populations of non-WT strains for TBF, global group and Chinese group, were significantly different, with the former up to 18.5% and the latter none. The percentages of isolates above UL-WT for ITZ in the two groups was low (global: 3.2%; China: 0).

**Table 3:** The minimum inhibitory concentration (MIC) and upper limit of wild type MIC (UL-WT) distribution of *T. rubrum i*solates against nine antifungal drugs tested using the CLSI M38-A2 broth microdilution method

Antifungal agent	No. of isolates	No. of isolates with MIC (mg/L)^a^ UL-WT																		UL-WT	% of NW	%of NW
		0.001	0.002	0.004	0.008	0.015	0.03	0.0625	0.125	0.25	0.5	1	2	4	8	16	32	64	95%	97.5%	95%	97.5%
LLCZ	92	10	1	3	16	**26**	17	16	2	1									0.0625	0.0625	3.3	3.3
	82^†^		1	3	16	**26**	17	16	2	1									0.0625	0.0625	3.7	3.7
BFZ	82^†^			1	2	19	**32**	23	2	1	1	1							0.125	0.125	3.7	3.7
MCZ	82^†^			1	2	9	20	17	**28**	3	2								0.5	0.5	0	0
KTZ	237					1	20	**46**	23	12	5	26	22						0.25	0.25	34.2	34.2
	132^†^					1	17	**41**	18	4	3	26	22						0.25	0.25	38.6	38.6
FCZ	85											25	**32**	12	12	2	1	1	8	8	4.7	4.7
	62^†^		0	0	0	0	0	0	0	0	0	25	**31**	6	0				8	8	0	0
ITZ	157					2	7	43	**62**	32	6	2	2	0	0	1			0.5	0.5	3.2	3.2
	112^†^					2	3	39	**47**	20	1								0.5	0.5	0	0
TBF	178	1	15	**39**	53	23	14	1	4	3	3	2	2	2	13	2	1		0.0312	0.0312	18.5	18.5
	132^†^	1	15	37	**53**	23	3												0.0312	0.0312	0	0
NAF	82^†^			1	8	42	**29**	2											0.0625	0.0625	0	0
AMF	82^†^					11	25	**39**	7										0.25	0.25	0	0

*NW* Non-wild type

Most frequently obtained MIC or mode is indicated in bold font

**“**†” Represents study from China

## Discussion

Dermatophyte infections have received renewed interest during the last five years because of the emergence of recalcitrant, highly virulent species in South Asia [19, 51]. The causative species was recently described as *T. indotineae* (*T. mentagrophytes* group) [52]. Given the rapid spread of dermatophytes, the potential replacement of mild *T. rubrum* by virulent *T. mentagrophytes* group is a significant public health risk. In China, *T. rubrum* is still the predominant species among dermatophytes, similar to previously published data [1, 3]. Antifungal resistance has also been reported in *T. rubrum* [16–19] and thus, a potential public health problem is apparent.

*T. rubrum* is identified phenotypically in the routine laboratory. For confirmation of identity, the rDNA internal transcribed spacer (ITS) barcoding gene is known to be sufficient for the distinction of siblings within the *T. rubrum* complex [27, 28]. In addition to 62 strains from our study, a global set of 71 sequences was found to be identical to *T. rubrum* haplotype H5 containing the type strain (CBS 392.58) [27]. The 100% match with phenotypic identification validates the IDs of older publications where no sequencing data are available. Five of the sequences, among which one from China, showed one SNP distance and clustered with *T. rubrum* H6 [27]. The ITS data support the earlier view of a global, largely clonal population structure with low levels of variation and no evidence of recombination [3]. The ITS marker is not epidemiologically associated with resistance, since 21 T*. rubrum* strains with higher MICs recorded in previous studies belonged to the same population [18, 19, 53], as confirmed in our study (Fig. 1).

Judging from proposed breakpoints for dermatophytes [49, 53], i.e., > 2 μg/mL for FCZ and > 1 μg/mL for ITZ, KTZ and TBF, the tested *T. rubrum* strains from China should be regarded as in vitro susceptible to all antifungal drugs. FCZ had a GM of 1.6172 μg/mL in our study, ranging from 1 to 4 μg/mL. These values are significantly lower than usually found in filamentous fungi (32 μg/mL; 54], but the compound is known to be ineffective in dermatophytosis [49]. In our study, MIC values against ITZ (GM 0.12 μg/mL, range 0.015–0.5 μg/mL) were relatively high. In contrast, the older azole KTZ demonstrated moderate activity with lower MIC values (GM 0.05718 μg/mL, range 0.015–0.25 μg/mL). TBF showed strongest activity (GM 0.0068 μg/mL) against *T. rubrum* in China. Comparing the activity of four drug categories based on chemistry and type of administration revealed statistically significant differences (Fig. 2B). Allylamines (TBF and NAF), which can be applied as topical or systemic agents, demonstrated stronger activity with lower MICs compared to remaining groups (*P* < 0.001). The topical azole category and the morpholines both had higher activity with lower MIC values in *T. rubrum* than the systemic azoles. In the terms of geography, differences between the Guizhou group and remaining Chinese provinces were not statistically significant for eight drugs. Only AMR had slightly higher MIC values in the Guizhou group than in remaining Chinese isolates. Since most strains (20/31) from Guizhou were isolated form toenails, all strains (31) in the China group were derived from foot skin; the reason for this difference needs further study. Nevertheless, the question of possible emerging resistance remains valid.

We therefore compared the MIC values in our study to similar studies in China as well as from other countries. Most of the English-language studies came from India (9/26) and Iran (6/26) (Table 2). Six additional studies from China were included. Although all studies were performed by following the CLSI M38-A2 protocol [29], small differences were observed in the protocols that we used, particularly in the incubation temperature: either 28 °C (six studies) or micro-broth dilution requiring incubation at 33–35 °C. It has been shown that 33 °C was the most suitable growth temperature for *T. rubrum* in terms of dry weight of mycelium and colony diameter [55]. The prevalence of *T. rubrum* involving protruding body parts is associated with an optimum growth below 35 °C [56]. The slow growth of the fungus requires reading of results after 5–7 days rather than after 72–96 h [57].

The most frequently studied drugs during the last decade globally are ITZ, FCZ, KTZ and TBF. Although the MIC range of each study is relatively large, the GM value of most antifungal drugs is fixed in a certain range, indicating repeatability and reliability of the research results, which lays a foundation for the interpretation of ECV of *T. rubrum* for different antifungal drugs. Among the four drugs, the GM values of FCZ are scattered between studies (Fig. 3e), and MIC values are relatively high. In contrast, MIC values against TBF and ITZ were consistent, and GM values in different studies were basically at the same level (Fig. 3f, g). However, two studies on TBF from India published in 2019, which indicated that *T. rubrum* isolates that are highly resistant to TBF, diverge significantly from remaining studies (Fig. 3g).

ECV analysis was performed simultaneously. We combined global distributions of MIC values from seven studies to distinguish between WT and non-WT populations and calculated upper limit of WT MIC (UL-WT) or epidemiological breakpoints. Since our study still could not totally fulfil the criteria of evaluation of ECV according to CLSI guidelines [58], we propose the UL-WT instead of ECV for *T. rubrum* of Chinese origin (Table 3).

FCZ was shown to have poor activity against *T. rubrum,* having the highest 97.5% UL-WT (8 μg/mL) agents. Different from *T. mentagrophytes / T. interdigitale* complex [59], the 97.5% UL-WT value of global *T. rubrum* for TBF was very low, i.e., 0.03 μg/mL. In contrast to China, the global group had a high percentage of isolates above UL-WT (global: 18.5%, China: 0), which indicated that TBF may be still considered as the first choice for treatment in China. Naftifine, another allylamine drug, also has good activity against *T. rubrum*, with a low 97.5% UL-WT (0.06 μg/mL). In addition to these two drugs, *T. rubrum* and *T. mentagrophytes/T. interdigitale* showed similar UL-WT values for the azole and morpholines [59]. LLCZ, BFZ and AMF are good options to treat *T. rubrum* infection due to low 97.5% UL-WT with a low percentage of non-WT isolates. However, the amount of strains tested for the above three drugs is small, and more verification is required. Based on the classification and comparison of antifungal agents in this study, we preliminarily determined that topical TBF and NAF should be still recommended as first-line therapy against superficial skin infection caused by *T. rubrum* in China. Antifungal creams should remain without steroids, and an adequate treatment period can be estimated at 2 weeks after the rash disappears [54], avoiding potential development of resistance. The azole drugs KTZ and MCZ, and particularly FCZ, are not recommended.

The limitation of this study is that the number of isolates is small; not every drug included MICs from the required minimum 100 unrelated isolates. However, our study preliminarily described and explored the UL-WT of *T. rubrum*, understood the trend of its sensitivity to a variety of antifungal agents and recommended first-line treatment for skin infection of this species in China. Regular surveillance of dermatophytes and antifungal susceptibility is recommended, since susceptibility profiles in general seem to be prone to change. At the same time, because of its highly conserved gene content, global prevalence and low virulence, *T. rubrum* may be a good choice as a research model for the mechanism of dermatophytes resistance.

## Supplementary Information

Below is the link to the electronic supplementary material.

Supplementary material 1 (DOCX 252 kb)
