# Supplementary material for: Regional Differences in Antifungal Susceptibility of the Prevalent Dermatophyte Trichophyton rubrum
Source: Mycopathologia. 2020 Dec 12;186(1):53–70. doi: 10.1007/s11046-020-00515-z (PMC7946697; doi:10.1007/s11046-020-00515-z)
Supplement: Supplementary file 1 — Supplementary material 1 (DOCX 252 kb) [file 11046_2020_515_MOESM1_ESM.docx]

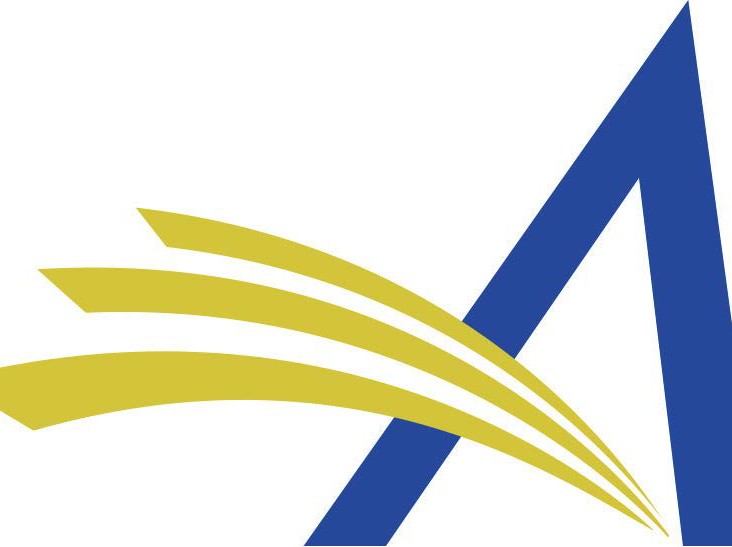

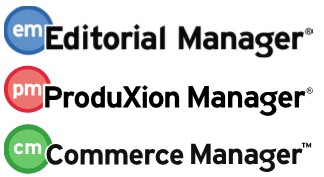


**Regional differences in antifungal susceptibility of the prevalent dermatophyte *Trichophyton rubrum***

**Y. Jiang ⋅ W. Luo ⋅ P.E. Verweij ⋅ Y. Song⋅ B. Zhang ⋅ Z. Shang ⋅ A.M.S. Al-Hatmi ⋅ S.A. Ahmed ⋅ Z. Wan ⋅ R. Li ⋅ G. S. de Hoog**

**Y. Jiang ⋅ W. Luo**

Department of Dermatology, The Affiliated Hospital, Guizhou Medical University, Guiyang, China

**Y. Jiang ⋅ P.E. Verweij⋅ S.A. Ahmed⋅ A.M.S. Al-Hatmi ⋅ G. S. de Hoog**

Department of Medical Microbiology, Radboud University Medical Center, and Center of Expertise in Mycology Radboudumc/CWZ, Nijmegen, The Netherlands;

**Y. Song (🖂) ⋅ Z. Wan ⋅ R. Li**

Department of Dermatology, Peking University First Hospital, Beijing, China;

Research Center for Medical Mycology, Peking University, Beijing, China;

National Clinical Research Center Research for Skin and Immune Disease, Beijing, China;

E-mail: syg3515@163.com;

**B. Zhang**

School of Public Health, Guizhou Medical University, Guiyang, China;

**Z. Shang**

Department of Immunology, Basic Medical School, Guizhou Medical University, Guiyang, China;

**S.A. Ahmed**

Faculty of Medical Laboratory Sciences, University of Khartoum, Khartoum, Sudan;

**A.M.S. Al-Hatmi**

Ministry of Health, Directorate General of Health Services, Ibri, Oman;

*#*Yanping Jiang and Wanglan Luo contributed equally to this work.

**Supplementary material**

**Table and figure supplementary legend**

**Table S-1.** MICs of the 62 *Trichophyton rubrum* isolates against 9 antifungals.

| Location(number) | Source | No.  of isolates | **Drugs MIC (μg/ml)** | | | | | | | | | | | | | | | | Genbank  accession numbers | |  |
| --- | --- | --- | --- | --- | --- | --- | --- | --- | --- | --- | --- | --- | --- | --- | --- | --- | --- | --- | --- | --- | --- |
|  |  |  | Azoles | | | | | | |  | | Allylamines | | |  | | Morpholines | |  |  |  |
|  |  |  | Topical | | |  | Systemic | | |  | | Systemic or topical | | |  | | Topical | | (ITS) | |  |
|  |  |  | LLCZ | BFZ | MCZ |  | KTZ | FCZ | ITZ | |  | | TBF | NAF | |  | | AMF | |  | |
| Guangzhou(5) | foot | 08616gz | 0.015 | 0.06 | 0.06 |  | 0.06 | 2 | 0.125 | |  | | 0.004 | 0.03 | |  | | 0.06 | | MW131245 | |
|  | foot | 08618gz | 0.015 | 0.03 | 0.06 |  | 0.06 | 2 | 0.125 | |  | | 0.004 | 0.03 | |  | | 0.06 | | MW131246 | |
|  | foot | 08619gz | 0.008 | 0.015 | 0.06 |  | 0.03 | 1 | 0.125 | |  | | 0.008 | 0.008 | |  | | 0.03 | | MW131247 | |
|  | foot | 08621gz | 0.03 | 0.25 | 0.5 |  | 0.25 | 4 | 0.5 | |  | | 0.015 | 0.03 | |  | | 0.125 | | MW131248 | |
|  | foot | 08622gz | 0.008 | 0.06 | 0.125 |  | 0.06 | 2 | 0.25 | |  | | 0.008 | 0.015 | |  | | 0.125 | | MW131249 | |
| Jilin(5) | foot | 08630jl | 0.03 | 0.06 | 0.06 |  | 0.06 | 2 | 0.125 | |  | | 0.015 | 0.03 | |  | | 0.06 | | MW131240 | |
|  | foot | 08631jl | 0.008 | 0.06 | 0.125 |  | 0.06 | 2 | 0.25 | |  | | 0.015 | 0.015 | |  | | 0.06 | | MW131241 | |
|  | foot | 08632jl | 0.06 | 0.06 | 0.125 |  | 0.125 | 2 | 0.25 | |  | | 0.008 | 0.015 | |  | | 0.03 | | MW131242 | |
|  | foot | 08634jl | 0.008 | 0.03 | 0.06 |  | 0.06 | 2 | 0.125 | |  | | 0.008 | 0.015 | |  | | 0.06 | | MW131243 | |
|  | foot | 08635jl | 0.008 | 0.125 | 0.25 |  | 0.125 | 2 | 0.25 | |  | | 0.008 | 0.015 | |  | | 0.06 | | MW131244 | |
| Xinjiang(5) | foot | 08638xj | 0.06 | 0.03 | 0.06 |  | 0.125 | 2 | 0.125 | |  | | 0.008 | 0.03 | |  | | 0.06 | | MW131297 | |
|  | foot | 08639xj | 0.03 | 0.015 | 0.015 |  | 0.03 | 1 | 0.06 | |  | | 0.002 | 0.03 | |  | | 0.03 | | MW131298 | |
|  | foot | 08640xj | 0.015 | 0.03 | 0.03 |  | 0.06 | 2 | 0.06 | |  | | 0.008 | 0.03 | |  | | 0.06 | | MW131299 | |
|  | foot | 08641xj | 0.015 | 0.03 | 0.25 |  | 0.03 | 2 | 0.25 | |  | | 0.008 | 0.03 | |  | | 0.03 | | MW131300 | |
|  | foot | 08642xj | 0.015 | 0.06 | 0.125 |  | 0.125 | 2 | 0.25 | |  | | 0.008 | 0.008 | |  | | 0.06 | | MW131301 | |
| Ningxia(6) | foot | 08674lx | 0.015 | 0.03 | 0.015 |  | 0.06 | 2 | 0.125 | |  | | 0.008 | 0.015 | |  | | 0.03 | | MW131291 | |
|  | foot | 08675lx | 0.03 | 0.03 | 0.03 |  | 0.03 | 2 | 0.125 | |  | | 0.008 | 0.008 | |  | | 0.06 | | MW131292 | |
|  | foot | 08676lx | 0.015 | 0.06 | 0.125 |  | 0.125 | 1 | 0.125 | |  | | 0.008 | 0.015 | |  | | 0.06 | | MW131293 | |
|  | foot | 08679lx | 0.015 | 0.03 | 0.125 |  | 0.06 | 1 | 0.03 | |  | | 0.002 | 0.015 | |  | | 0.03 | | MW131295 | |
|  | foot | 08678lx | 0.008 | 0.03 | 0.125 |  | 0.03 | 1 | 0.125 | |  | | 0.015 | 0.015 | |  | | 0.03 | | MW131294 | |
|  | foot | 08680lx | 0.015 | 0.06 | 0.015 |  | 0.06 | 1 | 0.03 | |  | | 0.004 | 0.03 | |  | | 0.06 | | MW131296 | |
| Sichuan(5) | foot | 08766sc | 0.06 | 0.008 | 0.125 |  | 0.03 | 1 | 0.125 | |  | | 0.015 | 0.03 | |  | | 0.06 | | MW131255 | |
|  | foot | 08767sc | 0.06 | 0.015 | 0.015 |  | 0.06 | 1 | 0.03 | |  | | 0.008 | 0.03 | |  | | 0.06 | | MW131256 | |
|  | foot | 08768sc | 0.06 | 0.03 | 0.125 |  | 0.06 | 2 | 0.125 | |  | | 0.015 | 0.03 | |  | | 0.03 | | MW131257 | |
|  | foot | 08769sc | 0.004 | 0.03 | 0.125 |  | 0.06 | 4 | 0.125 | |  | | 0.008 | 0.03 | |  | | 0.06 | | MW131258 | |
|  | foot | 08772sc | 0.015 | 0.06 | 0.125 |  | 0.06 | 2 | 0.25 | |  | | 0.008 | 0.015 | |  | | 0.06 | | MW131259 | |
| Wuhan(5) | foot | 08790wh | 0.004 | 0.03 | 0.03 |  | 0.03 | 1 | 0.06 | |  | | 0.002 | 0.03 | |  | | 0.03 | | MW131250 | |
|  | foot | 08791wh | 0.03 | 0.06 | 0.125 |  | 0.125 | 4 | 0.25 | |  | | 0.008 | 0.03 | |  | | 0.06 | | MW131251 | |
|  | foot | 08792wh | 0.03 | 0.015 | 0.03 |  | 0.03 | 1 | 0.06 | |  | | 0.015 | 0.03 | |  | | 0.03 | | MW131252 | |
|  | foot | 08793wh | 0.002 | 0.03 | 0.015 |  | 0.06 | 1 | 0.06 | |  | | 0.004 | 0.03 | |  | | 0.03 | | MW131253 | |
|  | foot | 08794wh | 0.015 | 0.03 | 0.004 |  | 0.03 | 1 | 0.25 | |  | | 0.004 | 0.015 | |  | | 0.015 | | MW131254 | |
| Guizhou (31) | foot | jyp18008 | 0.008 | 0.06 | 0.125 |  | 0.03 | 1 | 0.25 | |  | | 0.008 | 0.015 | |  | | 0.06 | | MW131261 | |
|  | foot | jyp18028 | 0.03 | 0.06 | 0.125 |  | 0.06 | 4 | 0.125 | |  | | 0.015 | 0.06 | |  | | 0.06 | | MW131262 | |
|  | foot | jyp18042 | 0.008 | 0.03 | 0.06 |  | 0.03 | 1 | 0.125 | |  | | 0.004 | 0.015 | |  | | 0.125 | | MW131264 | |
|  | foot | jyp18083 | 0.008 | 0.03 | 0.06 |  | 0.06 | 2 | 0.125 | |  | | 0.008 | 0.015 | |  | | 0.06 | | MW131277 | |
|  | foot | jyp18117 | 0.008 | 0.03 | 0.06 |  | 0.125 | 2 | 0.25 | |  | | 0.008 | 0.015 | |  | | 0.06 | | MW131286 | |
|  | head | jyp18054 | 0.015 | 0.03 | 0.03 |  | 0.06 | 1 | 0.06 | |  | | 0.008 | 0.015 | |  | | 0.06 | | MW131267 | |
|  | faciocervical | jyp19002 | 0.06 | 0.06 | 0.125 |  | 0.06 | 1 | 0.25 | |  | | 0.004 | 0.015 | |  | | 0.03 | | MW131287 | |
|  | plam | jyp18003 | 0.03 | 0.03 | 0.125 |  | 0.06 | 1 | 0.125 | |  | | 0.015 | 0.03 | |  | | 0.06 | | MW131260 | |
|  | groin | jyp18067 | 0.015 | 0.06 | 0.125 |  | 0.03 | 2 | 0.125 | |  | | 0.015 | 0.015 | |  | | 0.03 | | MW131272 | |
|  | groin | jyp19025 | 0.015 | 0.06 | 0.06 |  | 0.06 | 2 | 0.25 | |  | | 0.008 | 0.015 | |  | | 0.125 | | MW131288 | |
|  | hip | jyp18032 | 0.015 | 0.03 | 0.06 |  | 0.06 | 1 | 0.125 | |  | | 0.001 | 0.008 | |  | | 0.06 | | MW131263 | |
|  | toenails | jyp18050 | 0.015 | 0.03 | 0.06 |  | 0.015 | 1 | 0.015 | |  | | 0.002 | 0.015 | |  | | 0.06 | | MW131265 | |
|  | toenails | jyp18052 | 0.008 | 0.03 | 0.125 |  | 0.06 | 2 | 0.25 | |  | | 0.015 | 0.03 | |  | | 0.06 | | MW131266 | |
|  | toenails | jyp18055 | 0.03 | 0.015 | 0.06 |  | 0.125 | 1 | 0.06 | |  | | 0.002 | 0.015 | |  | | 0.06 | | MW131268 | |
|  | toenails | jyp18058 | 0.03 | 0.06 | 0.125 |  | 0.06 | 2 | 0.25 | |  | | 0.015 | 0.015 | |  | | 0.125 | | MW131269 | |
|  | toenails | jyp18060 | 0.03 | 0.06 | 0.125 |  | 0.06 | 2 | 0.25 | |  | | 0.015 | 0.03 | |  | | 0.125 | | MW131270 | |
|  | toenails | jyp18065 | 0.015 | 0.004 | 0.03 |  | 0.03 | 2 | 0.06 | |  | | 0.002 | 0.015 | |  | | 0.03 | | MW131271 | |
|  | toenails | jyp18068 | 0.06 | 0.03 | 0.015 |  | 0.06 | 2 | 0.06 | |  | | 0.008 | 0.015 | |  | | 0.06 | | MW131273 | |
|  | toenails | jyp18069 | 0.015 | 0.06 | 0.125 |  | 0.06 | 4 | 0.25 | |  | | 0.015 | 0.06 | |  | | 0.06 | | MW131274 | |
|  | toenails | jyp18072 | 0.008 | 0.03 | 0.06 |  | 0.06 | 1 | 0.125 | |  | | 0.008 | 0.015 | |  | | 0.06 | | MW131275 | |
|  | toenails | jyp18082 | 0.004 | 0.008 | 0.03 |  | 0.03 | 1 | 0.015 | |  | | 0.002 | 0.03 | |  | | 0.06 | | MW131276 | |
|  | toenails | jyp18085 | 0.06 | 0.015 | 0.008 |  | 0.125 | 2 | 0.125 | |  | | 0.008 | 0.03 | |  | | 0.06 | | MW131278 | |
|  | toenails | jyp18086 | 0.008 | 0.015 | 0.06 |  | 0.06 | 1 | 0.06 | |  | | 0.004 | 0.015 | |  | | 0.06 | | MW131279 | |
|  | toenails | 18087 | 0.008 | 0.015 | 0.06 |  | 0.06 | 2 | 0.125 | |  | | 0.008 | 0.03 | |  | | 0.06 | | MW131280 | |
|  | toenails | 18093 | 0.015 | 0.015 | 0.015 |  | 0.06 | 2 | 0.06 | |  | | 0.004 | 0.015 | |  | | 0.03 | | MW131281 | |
|  | toenails | 18094 | 0.06 | 0.125 | 0.125 |  | 0.125 | 4 | 0.25 | |  | | 0.015 | 0.03 | |  | | 0.06 | | MW131282 | |
|  | toenails | 18096 | 0.06 | 0.015 | 0.008 |  | 0.03 | 1 | 0.06 | |  | | 0.004 | 0.015 | |  | | 0.03 | | MW131283 | |
|  | toenails | 18112 | 0.06 | 0.015 | 0.03 |  | 0.03 | 1 | 0.125 | |  | | 0.015 | 0.03 | |  | | 0.06 | | MW131284 | |
|  | toenails | 18116 | 0.008 | 0.06 | 0.125 |  | 0.06 | 2 | 0.25 | |  | | 0.008 | 0.03 | |  | | 0.06 | | MW131285 | |
|  | toenails | 19043 | 0.015 | 0.03 | 0.03 |  | 0.06 | 2 | 0.125 | |  | | 0.004 | 0.015 | |  | | 0.03 | | MW131290 | |
|  | toenails | 19042 | 0.015 | 0.06 | 0.25 |  | 0.125 | 2 | 0.25 | |  | | 0.008 | 0.03 | |  | | 0.125 | | MW131289 | |
